# The role of duplications in the evolution of genomes highlights the need for evolutionary-based approaches in comparative genomics

**DOI:** 10.1186/1745-6150-6-11

**Published:** 2011-02-18

**Authors:** Anthony Levasseur, Pierre Pontarotti

**Affiliations:** 1 INRA, UMR1163 de Biotechnologie des Champignons Filamenteux, IFR86-BAIM. Universités de Provence et de la Méditerranée, ESIL, 163 avenue de Luminy, CP 925, 13288 Marseille Cedex 09, France; 2 Universités Aix-Marseille 1 et 2, UMR1163, 163 avenue de Luminy, CP925, 13288 Marseille Cedex 09, France; 3 Evolution Biologique et Modélisation.: UMR6632 CNRS, Université Aix-Marseille 1, 3 place V. Hugo, 13331 Marseille, France

## Abstract

Understanding the evolutionary plasticity of the genome requires a global, comparative approach in which genetic events are considered both in a phylogenetic framework and with regard to population genetics and environmental variables. In the mechanisms that generate adaptive and non-adaptive changes in genomes, segmental duplications (duplication of individual genes or genomic regions) and polyploidization (whole genome duplications) are well-known driving forces. The probability of fixation and maintenance of duplicates depends on many variables, including population sizes and selection regimes experienced by the corresponding genes: a combination of stochastic and adaptive mechanisms has shaped all genomes. A survey of experimental work shows that the distinction made between fixation and maintenance of duplicates still needs to be conceptualized and mathematically modeled. Here we review the mechanisms that increase or decrease the probability of fixation or maintenance of duplicated genes, and examine the outcome of these events on the adaptation of the organisms.

**Reviewers:**

This article was reviewed by Dr. Etienne Joly, Dr. Lutz Walter and Dr. W. Ford Doolittle.

## Background

Genomes are shaped by a series of processes involving substitutions, insertions, deletions, transpositions, shuffling of exons or chromosomes, lateral gene transfer, gene fusion or fission, *de novo *origination, and gene and genome duplications. The fate of the modified genes depends closely on the mutation type. For example, exon shuffling and substitutions are likely to have different outcomes on gene function and the subsequent putative fixation or maintenance of the new gene. Since the work of Ohno (1970) [1] gene and genome duplication has been considered as a primary driving force in the adaptive evolution of genomes and genetic systems. Duplication may even be considered as a "master mutation", as it promotes the accumulation of subsequent mutations on duplicates, as described below (substitutions, indels, etc.).

A duplication can be segmental (from a few nucleotides to several thousand kilobases) or may cover the whole genome (an event also called polyploidization). Segmental duplication (or small-scale duplication) and polyploidization correspond to distinct evolutionary processes with widely different impacts. Segmental duplication is a frequent event that occurs in all eukaryote lineages as part of a "continuous" process [2].

By contrast, polyploidization is a much more infrequent and spectacular mutation event that leads to either extinction or re-diploidization. The diploidization process, involving non-homologous recombination events together with deletions and pseudogenizations of genes, generates duplicated chromosomes that differ in large segments, but still exhibit paralogous regions.

Three polyploidization events have occurred in the last 150 million years in the *Arabidopsis thaliana *lineage. By contrast, no detectable polyploidization event has occurred in the *Drosophila *lineage for the last 600 million years. Segmental duplication of functional genes generates two copies, one of which generally loses its function rapidly through pseudogenization. Empirical data suggests that the majority of duplicates become pseudogenized in vertebrates. In the remaining cases, both duplicates are fixed either because one of the duplicates shifts toward a new function (neofunctionalization) or because the two copies subfunctionalize (*e.g.*, the copies differentiate their expression patterns through the evolution of different *cis-*regulatory modules).

The nature of the duplication event (segmental or whole-genome) will influence the likelihood of specific genes being lost or fixed. For instance, local duplication of a gene whose product is involved in a large interacting network will generate supernumerary duplicates that will disturb the network stoichiometry (gene dosage principle). In this case, the duplication event will be counter-selected. On the other hand, if the same gene is duplicated through a polyploidization event, all the members of the network will likewise be duplicated, retaining the network stoichiometry and reducing the likelihood of the duplicate being counter-selected.

In this review, the mechanisms of duplication, the processes of fixation or maintenance of duplicates and their impact in evolutionary history are discussed. We go on to describe experimental works that shed light on the fates of duplicates through classical events (such as neofunctionalization and subfunctionalization). Conceptual approaches are discussed that take into account evolutionary biology at the scale of gene, genome and population.

## Mechanisms of duplication

### Segmental duplication

The best-described mechanism causing segmental duplications from a few base pairs to several thousand kilobases is unequal crossing-over [3,4]. One of the most famous examples of large segmental duplication is the primate major histocompatibility complex (MHC) [5]. Other mechanisms that generate segmental duplications are transposition and retrotransposition events. As transposition is a "cut-and-paste" process, it is not likely that transposition results in a duplication events unless it is associated with lateral gene transfer (LGT). In specific cases, "cut-and-paste" transposition can result in duplication i.e. if it occurs in germ cells prior to meiosis and moves DNA from one chromosomal set to the other. LGT seems to be frequent only in bacteria and archebacteria [6], in which it can generate duplication. On the other hand, retrotransposition seems a frequent event in many eukaryote lineages. In metazoa, the maximum size of the duplicated segment corresponds to a transcript messenger (with the exclusion of *C. elegans*, in which co-transcription of multiple genes is possible).

### Whole genome duplication: polyploidization

This event is recurrent in eukaryotes (especially plants) and has also been described in bacteria [7-9]. In eukaryotes, documented polyploidization events comprise both autopolyploidy (polyploidization within a species) and allopolyploidy (hybridization between closely-related species). Polyploidization events that occurred long ago in the history of various lineages are difficult to detect because of subsequent remodeling of the genome through gene losses and recombinations. Ancient genome polyploidization (termed paleopolyploidization) has, for example, occurred in yeasts, angiosperms and teleost fishes [10]. Polyploidization can occur *via *various mechanisms, such as genomic doubling, gametic non-reduction and polyspermy. Genome doubling and gametic non-reduction involve failure of cell division during mitosis and meiosis, respectively. Unreduced eggs seem common in both animals and plants, whereas unreduced spermatozoa seem to be common only in plants.

An obstacle facing newly-formed tetraploid individuals is the fact that crossing with diploid relatives generates triploids, generally considered to be an evolutionary dead-end as they tend to produce aneuploid gametes owing to problems of chromosomal pairing and segregation during meiosis. However, it has been demonstrated that triploids can generate euploid (haploid, diploid, triploid) gametes at a low rate [11]. These euploid gametes can then produce triploid or tetraploid offspring. Thus triploids seem to be important, as they may facilitate the transition from diploidy to tetraploidy, and successful establishment of polyploidy appears to be facilitated by perenniality (because overlapping generations allow mating between triploids and their parents). Finally, selfing greatly facilitates polyploidization because it allows triploids to be maintained for several generations until stable polyploidy is generated. Interestingly, studies in plants have shown that the rate of polyploidy formation varies with environmental conditions and parental origin [12,13]. For instance, a sudden freeze during egg development causes more frequent production of unreduced gametes. High rates of hybridization (e.g., in hybrid zones) may facilitate polyploidization, which in turn facilitates the generation of isolated lineages, as polyploids tend to be reproductively isolated from diploid ancestors. Studies in plants have shown that the mean frequency of diploid gametes found in hybrids (28%) is about 50 times greater that in non-hybrids (around 0.5%). Interspecific hybrids often experience severe meiotic disorders because of homologous chromosome miss-pairing.

## Mechanisms involved in the fixation and maintenance of duplicates

### Conceptual distinction between fixation and maintenance of duplicates

Before going further, we need to make a conceptual distinction between fixation and maintenance of duplicates in order to decipher step-by-step the fates of duplicates in the genome. Numerous studies of gene duplication have focused on the mechanisms and functional consequences of duplicated genes at the molecular or organism scale. A biased interpretation of the role of duplication is carried out whether the process of duplication itself is not clearly unravelled. Three steps are responsible for leading to the generation of preserved gene duplicates: i) mutational events (duplication), ii) fixation of duplicates, and iii) maintenance or preservation. In this review, we define fixation rate as the probability that a duplicate, regardless of its functionality, spreads into a population (*i.e.*, becomes fixed), and maintenance rate as the probability that a duplicate is stabilized in a population (preservation). We can propose the following theoretical classification for gene duplicates:

Cat 1. Spreading difficult, maintenance difficult

Cat 2. Spreading difficult, maintenance easy

Cat 3. Spreading easy, maintenance difficult

Cat 4. Spreading easy, maintenance easy.

In the literature, duplication rate is a combination of the duplicating mutation and fixation rates. Most studies have used an empirical value for duplicating mutations (depending on specific lineages) and so the fixation rate is considered equal to the duplication rate. However, the rate of fixation of duplications cannot be used *a priori *to estimate rate of mutational origin [14]. Conceptual distinction between mutational generation (duplication), fixation and maintenance has critical implications for genome-scale studies as highlighted by experimental works. For instance, Davis and Petrov [15] investigated which types of genes are likely to generate functional and persistent duplicates and proposed that slowly evolving genes have a tendency to generate duplicates. Indeed, duplicated genes in the genomes of *Saccharomyces cerevisiae *and *Caenorhabditis elegans *have much slower rates of amino acid substitution, insertion and deletion than single copy genes. However, authors concluded that it is still unclear whether fixation, maintenance, or both of these steps together cause the bias towards the preferential duplication and highlighted that the relative importance of these two steps depends largely on the frequency with which duplicate genes are fixed by positive selection [15].

Duplication rate is also hard to estimate because of the difficulty in distinguishing true newly born duplicates from old ones that appear young because of gene conversion. Gene conversion is a homogenizing process between two homologous DNA fragments occurring during recombination. The divergence between two DNA fragments is biased and decreases dramatically following gene conversion. New models have been studied; for instance, Pan and Zhang propose an interesting strategy using unequal crossover and retrotransposition, to estimate rate that involves separate quantification of the rates of two different mechanisms of gene duplication and subsequent combination of the two rates, weighted according to their respective contributions to the overall gene duplication rate [16].

Conceptually, rate of duplication has to be considered as the resultant of a three-step process: duplicating mutations, fixation of duplicates, and finally maintenance of duplicates (long-term survival).

### Fates of genes after segmental duplication

Segmental duplication occurs in one individual within a breeding population, and the fixation of duplicates is constrained by classical variables of population genetics. Models of population genetics predict that an entirely redundant duplicate copy cannot be maintained in the genome for long, as harmful mutations will accumulate. Conversely, functional divergence will favor long-term retention of duplicates. Two major processes of divergence are possible; *(i) *neofunctionalization, where one copy retains the ancestral function while the other acquires a novel function ([1,17], and *(ii) *subfunctionalization, where the ancestral functions of the progenitor gene are partitioned between the duplicates, so that the union of activities and expression patterns of the duplicates are equivalent to those of the progenitor gene [18,19]. Modeling of the process predicts that subfunctionalization will be complex in populations with large effective sizes [20,21]. He and Zhang [22] broadened the concept of neofunctionalization by considering that a duplicate may retain all, none, or part of the ancestral functions. We note that different authors emphasize different meanings of 'gene function' [23]. For example, Hughes [18] refers to subfunctionalization of protein biochemical function, whereas Force *et al*. [19] emphasize subfunctionalized patterns of gene expression.

One model, the duplication-degeneration-complementation model (DDC) describing the fate of duplicates was proposed by Force *et al*. [19] and illustrated in the works of van Hoof [24]. It involves complementary degenerative mutations in *cis*-regulatory modules: a fixed degenerative mutation in a regulatory module of duplicate A is followed by *(i) *accumulation of additional fixed degenerative mutations in the same copy, leading to its pseudogenization, or *(ii) *mutations, in copy B, of a complementary regulatory module (this second mutation occurs in a module that remains intact in copy A so that the two copies become essential for complete gene expression, preventing pseudogenization of either one), or *(iii) *by acquisition of a new function in copy B, through mutation in a complementary regulatory module (copy A is retained because it exhibits the original function associated with the non-mutated regulatory module). Obviously, probabilities of fixation of duplicates will also depend on the size of the population, and the selective pressures associated with the mutations in the corresponding *cis*-regulatory modules.

### Fate of genes after polyploidization

One striking result concerning polyploids is that despite unstable genomes and rapid re-patterning, the addition to the genome of a complete set of chromosomes is remarkably well-tolerated in eukaryotes (and many current species descend from polyploid ancestors). Why is whole genome duplication more likely to generate lineages that persist over evolutionary time? Although the fate of genes after whole genome duplication depends on mechanisms similar to those discussed above for local duplication (pseudogenization, neofunctionalization and subfunctionalization), fixation of polyploidy cannot be discussed solely in the same terms as fixation of segmental duplicates, because polyploids tend to be at least partially isolated from the ancestral (non-polyploid) population by reproductive incompatibility. Furthermore, in the case of plants, polyploids can often be maintained through selfing and vegetative reproduction.

We might expect the offspring of polyploid individuals to be necessarily polyploid. However, experiments on synthetic polyploids show that gene inactivation or subfunctionalization occur as early as the first generation *via *genome imprinting and genomic changes [25]. This process has been evidenced for both allo- and autopolyploids [26]. Song *et al*. [25] observed extensive genomic rearrangements and fragment losses within five generations of plant hybrids in the genus *Brassica*. Other studies report genomic changes soon after formation of wheat and *Arabidopsis *allopolyploids but not in cotton or cordgrass *Spartina *(a natural polyploid) [27]. In most of the examples studied, rapid genomic re-patterning has been observed in allopolyploids but not in autopolyploids. There are several reasons to suppose that hybridization may be responsible for re-patterning. For instance, transposable elements that are repressed within each parent lineage, but that can be activated in hybrids, could facilitate gene translocation and unequal crossing-overs. Josefsson *et al*. [28] found that maternally-derived siRNAs of hybrids were not sufficient to repress the retrotransposons originating from the parental genomes in *Arabidopsis thaliana *X *Arabidopsis arenosa *hybrids. Furthermore, divergence between centromeric histones from parental species may lead to chromosome segregation distortion and non-disjunction in hybrids. In addition, non-homologous recombination and non-reciprocal exchanges are particularly likely among homologous chromosomes with structural differences. Nevertheless, genomic re-patterning in polyploidy is not driven exclusively by hybridization. In autotetraploids of both *Candida albicans *[29] and *S. cerevisiae *[30], genome size reduction through chromosome losses has been observed. In the second study, haploid and tetraploid lines reverted to diploidy in 1800 generations. These experiments show that entire sets of chromosomes can be lost, although the exact mechanisms involved remain unknown.

Genomic re-patterning may also increase the genetic variability of newly formed polyploid populations. This variability can be beneficial for the generated polyploid lineage as it can counteract the reduction of variability due to drastic reduction of population size, which is generally associated with polyploidization events. Surviving polyploids therefore probably form a biased subset of those that have been generated; we witness only lineages that have evolved towards particularly fit and stable genomic configurations soon after polyploidization.

Besides drastic structural changes in their genomes, polyploids also often exhibit tissue-specific changes in gene expression (for review see [27]). This is especially so for allopolyploids, which can experience *(i) *changes in methylation [31], *(ii) *disruption of heterochromatin leading to retrotransposon activation [28] and *(iii) *alteration in imprinting and biased expression of homologs [32]. For example, Adams *et al*. [26] found that cotton allopolyploids differed from parental individuals in tissue-specific expression patterns for 11 out of 18 genes analyzed.

As is the case for genome rearrangement, most changes in gene expression seem to be due to hybridization rather than to polyploidy *per se *[31,33] and are correlated with divergence between the parental species [28]. Much smaller effects on gene expression were found in autopolyploids than in allopolyploids. Proteomic analyses in autopolyploid cabbage have shown very few expression changes [34]. Song *et al*. [25] also observed that less extensive genomic rearrangements occurred in allopolyploids when formed from more closely related species.

### Mechanisms favoring or opposing fixation or maintenance of duplicates

#### Neofunctionalization

Neofunctionalization is context-dependent and may require multiple mutations. This process is better known as the 'Dykhuisen-Hartl' effect [35,36]: as one copy of a duplicated gene can freely mutate (a single copy must remain under pre-existing selective constraints), these mutations can lead to either pseudogenization or neofunctionalization. Mutations that are neutral in a particular environment can be positively selected by new environments or by epistatic interactions with subsequent mutations. The coefficient of selection can therefore vary in time.

Neofunctionalization can be classified into two types: "*stricto sensu" *neofunctionalization and micro-neofunctionalization. The first type involves a radical shift in biochemical function or expression pattern, giving rise to a new function at high levels of organization [37,38]. Examples in the literature include the crystalline proteins, the antifreeze proteins and many proteins from the major histocompatibility complex (MHC) (for a review, see [23,37].

The second type, namely micro-neofunctionalization, involves a shift in the specificity of a metabolic activity, or in affinity for a given ligand, etc. [39]. Genes involved in recognition of the environment (such as olfactory receptors or MHC genes) probably originated through micro-neofunctionalization.

Micro-neofunctionalization may be responsible for the surprising observation that bacterial strains can be recovered at higher-than-expected frequencies when they are plated on specific media in which mutations are advantageous. Hendrickson *et al*. [40] showed that increased mutation frequency was the direct consequence of an increase in the target gene copy number. For example, bacteria carrying a defective, but leaky, *lacZ*^- ^allele produced more *lacZ*^*+ *^revertants than expected when cultures were plated on lactose minimal medium. Most of these mutants appeared not during growth in liquid medium (*i.e.*, before plating), but after a period of very slow growth on the lactose plates. The mechanisms responsible for this effect are as follows: first, the *lacZ*^- ^allele is strongly expressed, allowing bacteria to survive on lactose by producing very large amounts of the defective enzyme; this increase in gene expression is selectively advantageous, as it amplifies the minimal activity of the *lacZ*^- ^allele to a level that permits cell survival. Second, the presence of multiple copies of the *lacZ*^- ^allele makes more likely the chance occurrence of a mutation restoring the wild type (LacZ^+^) activity, and thus optimal growth on lactose. Once a gene copy reverts to the wild type allele, it spreads throughout the bacterial population and overruns the other gene copies, which rapidly disappear. This model proposed by Francino [41] is called 'adaptive radiation'. It postulates an initial period of positive selection for gene amplification, followed by positive selection on the paralog copies for the acquisition of an advantageous phenotype. As proposed in the *lacZ *system, gene amplification could initially provide the means to reach biologically relevant levels of protein functionality, before neofunctionalization occurs. The evolution or expansion of multigenic families involved in sensory perception (*e.g.*, olfactory receptor families) may be partly explained by this process.

#### Subfunctionalization *via *specialization

Several authors have shown that neither neofunctionalization nor subfunctionalization alone can adequately account for retention. Analysis of the genome-wide patterns of yeast protein interaction and human gene expression for duplicate genes has revealed rapid subfunctionalization accompanied by prolonged, substantial "*stricto sensu*" neofunctionalization in a large proportion of duplicate genes, suggesting a new model, termed "sub-neofunctionalization^"^. A possible biological explanation is that subfunctionalization could be followed by neofunctionalization with positive selection because of the pleiotropic constraint release [18,22,42]. However, we could also consider that the term "specialization" would be more appropriate in this particular case.

#### Gene duplications and genetic robustness

Genetic robustness can be defined as *(i) *the ability of a biological system to withstand mutations due to redundancy, here the ability of duplicates to balance loss of function in other copies and *(ii) *participation in a biological network, *e.g.*, alternative metabolic pathways and regulatory networks. Duplicate genes undergo relaxed selection shortly after their duplication, which enables them to tolerate more nucleotide changes than their single-copy counterparts. Similar scenarios take place for whole genome duplication (WGD), where gene duplicates can tolerate up to 10 times more amino acid changes than old duplicates in vertebrates [43,44]. Robustness is therefore essential in evolutionary innovation and phenotypic diversity.

#### Dominant negative mutations

Dominant negative mutations are mutations whose gene product adversely affects the function of the normal wild-type gene product within the same cell. Dominant negative mutations are therefore often more damaging than null mutations. The probability of gene loss will therefore be correlated with the proportion of possible dominant negative mutations. Cooke *et al*. [45] has hypothesized that partial protein damage has a stronger phenotype than null mutants caused by loss of gene expression. It has been stated above that duplicated genes with full redundancy can be expected to reduce to a single copy over time through the stochastic accumulation of mutations that harm one of the genes. In some genes, point mutations damaging protein integrity could also cause a defective phenotype. Thus the genetic redundancy cannot easily decay away through the accumulation of point mutations. The corollary of this is seen in knock-out studies showing that many genes can be removed in a single step that abolishes the expression of the protein-encoding gene, although point mutations of these proteins often have phenotypes. Cooke argued that naturally-occurring deletions are rarer than point mutations and to be viable must avoid damaging neighboring genes. Hence only a few deletion events suffice to knock out a gene. This effect will increase according to the interactiveness of the protein. Therefore, highly connected protein duplicates are retained because of gene dosage sensitivity and dominant negative counter-effect.

#### Dosage sensitivity: dual consequences

If the product of a duplicated gene belongs to a large protein complex, the duplication event can be counter-selected because it may generate imbalance among members of the protein complex. The gene dosage balance hypothesis (GDBH) proposes that such stoichiometric imbalances in macromolecular complexes are a source of dominant negative phenotypes [46,47]. General evidence supporting the GDBH has been found for example in yeast and *Arabidopsis thaliana*: focusing on essential genes, Papp et al. [48] have shown that dosage-sensitive genes are at least twice as likely to encode proteins involved in complexes as genes with low dosage sensitivity. Furthermore, a statistically significant higher proportion of genes whose overexpression is lethal encode proteins involved in complexes [48]. Three predictions, largely confirmed by experimental work, can be made: *(i) *artificial overexpression of one subunit should be harmful, *(ii) *the strength of transcriptional co-regulation of subunits can be expected to reflect dosage sensitivity, and *(iii) *duplication of a single gene whose product is involved in a protein complex is likely to be harmful. Besides protein interaction stoichiometry, gene balance is believed to be governed by regulatory effects. Relative numbers of regulatory genes modify the expression of the target genes. Whenever there is competition between different offspring, dosage-sensitive gene losses will be counter-selected. Dosage sensitivity can be qualified by a dosage compensation effect. A *trans*-acting dosage effect can negatively affect the expression not only of genes located elsewhere in the genome, but also of the genes present on the same chromosome, yielding a compensation result. By contrast, duplication by WGD increases the dosage of all genes and so should not affect the balance. Analysis of duplicate genes arising from paleopolyploidization events in angiosperm, vertebrate, teleost, yeast and *Paramecium *phyla [49] support this prediction: the transcription factors along with proteins involved in protein binding, protein modification, and protein degradation, were more strongly retained than other protein functional classes [46-48].

#### Putative role of hitchhiking in gene duplication

Local duplication can involve large genomic regions encompassing several genes. The probabilities of neofunctionalization, subfunctionalization or pseudogenization of each gene in the duplicon are unchanged, but positive or negative selection on a gene within the duplicon will influence the fate of the linked genes [50].

#### Intrinsic genome evolution

Lynch and Conery [2] suggested an inverse correlation between population size and genome size (the difference in genome sizes among species being due to intron size, the presence of different repetitive elements, and the presence of duplicates). They suggested that purifying selection was intense in large populations, essentially precluding fixation of significantly damaging mutations, whereas mutation with substantial damaging effects could be fixed by random drift in small populations. Hence duplications might be fixed despite their potentially damaging nature. For instance, a newly inserted intron requires a critical mass of nucleotides (*n *= 20-40 nucleotides in range) to be accurately recognized and removed. Understanding the origins of eukaryotic genome complexity in adaptive terms is rendered difficult by the fact that each length increase of a gene raises its vulnerability to mutational inactivation, thereby favoring its elimination from the population.

## Relative contributions of polyploidization *versus *local duplication to genome evolution

If duplications are considered as a major source of genomic novelty, then the frequency of such events will be crucial to the evolution of species. Participation in the creation of new genetic materials from whole genome duplication and local duplication will depend on the phylum. Some phyla show greater propensity than others to be polyploidized. For instance, the angiosperms contain 30 to 80 percent of species in a neopolyploid state, unlike the *Drosophila *lineages, which do not seem to have polyploidized for at least 600 million years. Regional duplication processes will probably have a deeper impact in species that do not polyploidize than in those that do. Around 15% of the genes in the human genome are believed to arise from duplication events, whereas gene duplicates account for 8-20% of the *Drosophila melanogaster*, *Caenorhabditis elegans*, and *Saccharomyces cerevisiae *genomes. However, these estimates are highly dependent on the sensitivity required to determine when a duplicate is detectable and others works hypothesized that almost all human genes resulted from ancient duplication [51].

### Examples in vertebrates

Genomic comparative analyses have revealed unexpected dynamics concerning family size, and once again underline the importance of gene and genome duplication in the history of evolution. In vertebrates, two rounds of whole genome duplications are thought to have played an essential role in the establishment of gene repertoires [52]. These events occurred during chordate evolution after the split of the urochordate and cephalochordate lineages, before the radiation of extant gnathostomes (jawed vertebrates). The rate of local duplication is estimated at between 1 gene per 100 and 1 gene per 1000 per million years [44,53].

Calculations were performed as described below. Lynch and Conery assumed that the number of silent substitutions per site increased approximately linearly with time. The relative age distribution of gene duplicates within a genome can therefore be inferred indirectly from the distribution of silent substitutions. For all the species tested the highest density of duplicates tended to be contained within the youngest age classes, with the density dropping off rapidly with increasing silent substitution. A smooth decay was seen with species that had probably not recently polyploidized, such as *Homo sapiens*. This observation is explained by the birth and death process [54]; the youngest age category represents newly arisen duplicates, and the subsequent decline in frequency results from mutational processes that eliminate complete open reading frames (deletions or frameshift mutations). The rates of birth and loss of such genes can be derived directly from the observed age distribution, assuming that these rates have remained essentially constant within the age class employed in the analysis. From this analysis Lynch and Conery found that the average probability of duplication of a eukaryotic gene was 1 percent per million years.

Cotton and Page also showed that a constant birth and death rate model was appropriate for gene duplication data, allowing the estimation of the rate of gene duplication and loss in vertebrates over the last 200 Myr (0.115 percent duplication, 0.74 percent losses). In this case, they used estimated times from fossils and molecular clock data. We must bear in mind that the aim of such analyses is to evaluate the average evolutionary properties of the members of duplicate gene pairs, and that some of the gene pairs will survive longer than average owing to positive selection (*via *neofunctionalization and subfunctionalization) or be shorter-lived than average owing to negative selection against the duplicate (see above).

### Dynamics of gene family size

The comparison of whole genomes reveals changes in the size of specific gene families among organisms [55], and several authors have found it possible to infer ancestral state and deduce which lineages in gene families have contracted or expanded. This approach enables us to classify gene families into the conceptual categories listed in III.1. The authors used a model of stochastic birth and death for the gene family that could be applied to multispecies genome comparisons. This model takes into account the branch length of phylogenetic trees, together with duplication and deletion rates, and so provides expectations for divergence in gene family size among lineages. This affords an estimate for the rate of fixation and loss for a given family. The analysis of Hahn *et al*. [55] is based on birth and death processes, but it might be more usefully considered as a fixation index and maintenance index. They analyzed gene families contained within the whole human genome, chimpanzee, mouse, rat and dog, and found that more than half of the 9990 families present in the mammalian common ancestor had either expanded or contracted along at least one lineage. They also found 164 families to be evolving non-randomly at *P *< 10^-5^. With this cut-off threshold, they expected no family to be significant by chance. The most common biological functions assigned to these gene families included immune defense gene, neuron developments and intercellular communication and transport. Interestingly, comparisons of both synonymous to non-synonymous nucleotide divergence and regulatory sequence divergence also showed gene categories with these biological functions. The authors concluded that natural selection could act at many levels during adaptive molecular evolution. The real situation could probably be more complex, as a duplicate that is not important for the function will tend to be less constrained (some proteins are more constrained than others; environmental proteins may have less constrained sites). Specific functional studies on these families would thus be informative.

## Experimental work

### Non-evolutionary-biology-based analyses

Numerous analyses based on non-evolutionary approaches have been published. Below we describe two interesting examples that demonstrate the need to integrate evolutionary history into experimental work.

#### Interactome analyses

He and Zhang [22] analyzed the high confidence interaction data compiled by Von Mering and those annotated in the MIPS database. A total of 331 gene pairs and 745 singleton genes underwent the following analysis: the authors looked for duplicate pairs, numbers of specific partners and numbers of shared partners. After gene duplication the two duplicates have the same interaction partners. In the subfunctionalization model, each duplicate gradually loses partners, but the number of total partners remains constant over time. The mean number of total partners for duplicate genes was about 8.6. This was more than for a singleton gene, where a value of 4.7 was found (the difference was statistically tested). The authors concluded that the model that best explained their results was the neofunctionalization model. However, it is still possible that genes staying in single copy and duplicating genes have different number of partners, and evolutionary-based analysis should be performed to conclude.

#### Expression analyses

He and Zhang also analyzed human gene expression including the expression levels of 7565 human genes in 25 independent and non-redundant tissues [56]. They transformed the quantitative expression levels into discrete expression patterns (expressed or unexpressed). They analyzed expression patterns of 515 singletons and 1230 pairs of duplicate genes and found that the number of expression sites per duplicate pair was significantly greater than that per singleton gene. This refutes the pure subfunctionalization model. Using the synonymous mutation distance between duplicates as a clock, they examined how the number of expression sites had increased over time since duplication. To reduce random fluctuations, they put duplicates into seven bins depending on their divergence times and found that the number of expression sites and the times of duplication were positively correlated.

### Evolutionary-biology-based analyses

Ideally, all studies concerning the function of duplicates should be integrated into evolutionary-based approaches where the history of a gene and the corresponding function have to be sought. Phylogenetic methods, for example, have been developed for inferring ancestral expression profiles or ancestral functions of homologs. In this case, the whole family history has to be integrated using the information at each branch of the tree in order to deduce the ancestral pattern or ancestral function at each node. Thus inferences about nodal values permit the estimation of evolutionary changes along each branch segment of an evolutionary tree [57,58]. Because information is scant, authors use "trio information", *i.e.*, between one ortholog and two co-orthologs. In this case, the authors assume that orthologs retain the ancestral expression or function.

#### Gene-centred analyses

##### Subfunctionalization

Hittinger and Caroll [59] investigated the evolution of one pair of duplicates in *S. cerevisiae*: GAL1 and GAL3. GAL1 encodes the galactokinase enzyme and the GAL3 gene encodes a co-inducer of galactokinase, able to sequester a repressor of the gene transcription factor activating the galactose use pathway. GAL1 and GAL3 have a co-ortholog in *Kluyveromyces lactis*. Phylogenetic analysis showed that GAL1/GAL3 duplicated in the *S. cerevisiae *lineage after the *K. lactis *and *S. cerevisiae *split. Compared with the *K. lactis *co-ortholog, GAL3 has lost its enzymatic activity, whereas GAL1 has changed its regulatory requirements. The outcome is a more tightly controlled and more highly inducible GAL1. The authors tested whether GAL1 and GAL3 duplication and subfunctionalization could have been fixed *via *positive selection and tested fitness differences in genetically manipulated *K. lactis*. Increased expression of the gene module providing galactokinase activity enhanced fitness, whereas overexpression of the module equivalent to GAL3 reduced fitness. In *S. cerevisiae*, there is no such conflict, and therefore the subfunctionalization could have been positively selected.

##### Subfunctionalization and genetic robustness

Hickman and Rusche [60] studied the duplicated histone deacetylases Sir2p and Hst1p in *S. cerevisiae *and found that these paralogs with non-overlapping functions could confer genetic robustness against null mutations through a substitution mechanism. Hst1p is a NAD(+)-dependent histone deacetylase that acts with Sum1p to repress a subset of mid-sporulation genes. However, the mutant deleted for hst1 showed much weaker derepression of target loci than the mutants deleted for sum1. The authors showed that this weak derepression of target loci in hst1Delta strains occurs partly because Sir2p substitutes for Hst1p. Sir2p helps to repress the mid-sporulation genes only in the absence of Hst1p and is recruited to target promoters by a physical interaction with the Sum1 complex. Also, when Sir2p associates with the Sum1 complex, the complex continues to repress in a promoter-specific manner and does not spread. In addition, SIR2/HST1 gene from *Kluyveromyces lactis*, a closely related species that diverged prior to the duplication, can suppress an hst1D mutation in *S. cerevisiae *as well as interact with Sir4p. These results suggest that the evolutionary path of duplicate gene preservation may be an important indicator for the ability of duplicated genes to contribute to genetic robustness.

##### Subfunctionalization deduced from protein architecture

Cusack and Wolfe describe how a bifunctional gene, encoding two proteins by alternative splicing, arose when the chloroplast gene RPL32 integrated into an intron of the nuclear gene SODcp in an ancestor of mangrove and poplar trees [61]. Mangrove retained the alternatively spliced chimeric gene, but in the poplar lineage, it underwent duplication and subfunctionalization, through complementary structural degeneration, to re-form separate RPL32 and SODcp genes. The partitioning process is considered to be a subfunctionalization because structural changes in the poplar genes indicated that after duplication a complementary loss of subfunctions of the ancestral chimeric gene occurred in its two daughter genes. The losses of exon X (encoding the RPL32 subfunction) in the *Poplar2 *and *Poplar3 *lineage, and of exons 4, 7 and 8 (encoding the SOD subfunction) in *Poplar1*, were caused by degenerative mutations that were probably selectively neutral because in each case the subfunction lost by one gene copy was maintained by the other.

##### Neofunctionalization with functional evidence

Zhang [62] reports that the gene encoding pancreatic ribonuclease was duplicated independently in Asian and African leaf-eating monkeys. Statistical analyses of DNA sequences, functional assays of reconstructed ancestral proteins and site-directed mutagenesis showed that the new genes acquired enhanced digestive efficiencies through parallel amino acid replacements driven by positive selection. They also lost a non-digestive function independently, under a relaxed selective constraint. These results demonstrate that despite the overall stochasticity, even molecular evolution has a certain degree of repeatability and predictability under the pressures of natural selection.

#### Large scale analyses

Growing information resulting from DNA sequence data enable us to carry out large-scale comparative analyses.

##### Indirect information about biochemical function

Scannell and Wolfe [63] studied genes for which either a single copy ortholog or double copy co-orthologs were available in eight yeast species (four of which diverged post-WGD while the four others diverged from an ancestor pre-WGD). They showed that, on average, proteins encoded by duplicate pairs evolved at least three times faster immediately post-WGD than single copy genes, to which they behave identically in non-WGD lineages. Although the high rate of duplicated genes subsequently declined rapidly, it has not yet reverted to the typical rate for single copy genes. They also showed that although duplicate gene pairs often have highly asymmetric rates of evolution, even the slower members of pairs showed evidence of bursts of evolution after duplication. Asymmetry after duplication was also evidenced in teleosts [64]. Kellis *et al*. demonstrate that the yeast *Saccharomyces cerevisiae *arose from ancient WGD, by sequencing and analysing a close specie *Kluyveromyces waltii*. Their results provide the first comparison across an ancient WGD event and offer the opportunity to study the long-term fate of a genome after duplication. In the majority of cases (95%), accelerated evolution concerned only one of the two paralogues. These results strongly support the model in which one of the paralogues retained an ancestral function while the other, relieved of this selective constraint, was free to evolve more rapidly [65]. This asymmetry could reflect positive selection or relaxation leading to neofunctionalization or subfunctionalization.

##### Expression and functional shift analyses

Tirosh and Barkai [66] developed a method to compare expression profiles from different organisms and applied it to analyze the expression divergence of yeast duplicated genes. Expression profiles of *S. cerevisae *duplicate pairs were compared with those of their co-orthologs in *C. albicans*. Duplicate pairs were divided into two classes: symmetric *versus *asymmetric rates of expression divergence. The expression of many of these duplicate pairs is highly correlated, suggesting that they were retained by selection for high protein dosage or evolved through other functional aspects such as protein structure or interaction. The asymmetric class includes 43 duplicate gene pairs in which only one copy showed a significant expression similarity to the *C. albicans *ortholog. Some of these cases may involve neutral evolution of gene expression of no functional significance, or they may involve regulatory neofunctionalization.

Wapinski *et al*. [49] developed a procedure to resolve the evolutionary history of all genes in a large group of species. Their procedures were applied to 17 fungal genomes to create a genome-wide catalog of gene trees to determine precise orthology and paralogy relationships across these species. Gene duplication and loss are highly constrained by the functional properties and interaction partners of genes. Annotations were performed with the well-annotated *S. cerevisiae*. In particular, stress-related genes exhibited many duplications and losses, whereas growth-related genes showed selection against such changes. Whole genome duplication circumvents these constraints and relaxes the dichotomy, resulting in an expanded functional scope of gene duplication. By characterizing the functional fate of duplicate genes, they showed that duplicated genes rarely diverged with respect to the biochemical function, but typically diverged with respect to regulatory control. Gene duplication may drive the modularization of functional network through specialization, thereby disentangling cellular systems. Earlier observations suggested that paralogous modules were formed in massive duplication events. Wapinski *et al*. found that paralogous modules were rare even post-WGD and suggested an alternative mechanism. Many paralogous pairs genetically interact with each other despite having no shared physical interactions, which may induce a partial division of labor (subfunctionalization) between two paralogous proteins that become physically or temporally separated. Such specialization could modularize a molecular network by separating links within a network when duplicating a node. Thus increasing gene copy number may simplify a system rather than making it more complex. Modularization could relax opposing constraints on a single component and thus set in motion further specialization and refinement [49]. This report compares functional behaviour at different levels between duplicated genes, and shows that gene duplication innovates through regulatory divergence.

After duplication of several genes, these can either migrate in a coordinated manner, resulting in two paralogous classes, or be dispersed into different classes. The authors expected coordinated migrations after simultaneous duplications. To test this hypothesis, they counted the number of paralogous gene pairs connecting each pair of gene classes (transcriptional classes, biochemical classes, etc.) and found that coordinated migration was rare. Gene classes (functional regulatory or transcriptional) rarely shared more than one or two paralogous relations regardless of the overall proportion of retained paralogs. The few observed paralog classes are very small and were formed gradually (from independent duplications). Thus paralogs dispersed individually.

##### Subcellular localization shift

Marques *et al*. [67] analyzed the possibility of neofunctionalization or subfunctionalization in subcellular localization. In their studies, the authors used first a non-evolutionary-based approach, and then an evolutionary-based one. The first non evolutionary-based approach hypothesized that divergent subcellular localization between duplicates was a consequence of sublocalization (subfunctionalization) alone. The joint number of different compartments per protein pair (combining both duplicates) would be expected to be the same as that of the common ancestral protein. Conversely, the number of compartments per pair should be higher than that of the progenitor if neo-localization contributed to sub-cellular diversification. However, sublocalization data for ancestral protein or for an outgroup were lacking. Thus to assess the contribution of neo- and sublocalization to the functional diversification of duplicates, the authors used the "He and Zhang" strategy. They used the average number of subcellular compartments of yeast singleton gene products as a proxy for the subcellular representation of WGD duplicate progenitors of yeast duplicates. They observed that the joint number of distinct compartments per WGD-derived duplicate with distinct cellular localization was significantly higher than that observed for singleton proteins. By contrast, there was no difference between the distributions of the number of subcellular compartments for WGD duplicates with the same subcellular distribution. This suggests that the increase in the number of compartments observed for the WGD-derived pair with distinct cellular localization was due to neolocalization events among these duplicates. The authors underline that their conclusions require a caveat: the types of proteins represented in the WGD-derived pair with distinct cellular localization may generally and *a priori *be present in a larger number of compartments. To check this, they compared the number of distinct compartments per WGD-derived pair with distinct cellular localization and singletons for proteins within the same Gene Ontology (GO) classes. This analysis showed that for all the GO classes tested, the joint number of compartments per WGD-derived pair with distinct cellular localization was significantly higher than that observed for singletons. This suggests that the elevated number of compartments for D-pairs (WGD-derived duplicates with distinct cellular localization) could be the result of neolocalization and not of a wide cellular representation of ancestral progenitor proteins, prior to duplication. They also used an evolutionary-based approach on a few families where the functional information was available in sister species (*K. waltii*). They then constructed the phylogeny for 45 yeast families, mapped the subcellular localizations of these onto the phylogenies and finally used a parsimony-based analysis to deduce the ancestral and derived states (function). In 16 families, the subcellular localization has remained fully preserved among members. For the remaining 29 families, they analyzed changes in protein location, assuming that the scenario requiring the smallest number of subcellular changes, given the observed data (parsimony principle), reflected the true pattern of events. For 16 of the 29 families, they inferred the most likely scenario of subcellular diversification. Eight families showed instances of neolocalization. For example, members of the ubiquitin-conjugating enzyme family, involved in protein degradation, are generally located in the cytoplasm and the nucleus.

## Conclusion

In the present review, we have discussed genome evolution *via *duplication and the mechanisms involved in the fixation and maintenance of the duplicates. The forces driving the fates of duplicate genes rely not only on duplication type (*i.e.*, segmental duplication or whole genome duplication), but also on several phenomena (opposing or compensatory) linked to population size, gene function and gene balance. Commonly, local duplication may be eliminated by passive losses following the genetic population laws, whereas in the case of whole genome duplication, duplicates or chromosome losses are an active, complex biological process resulting from an equilibrium disturbance in the cell.

To decipher the role and impact of duplication in genome evolution, future works could be usefully reinforced in the following main directions:

a) The distinction between fixation and maintenance of duplicates needs to be biologically conceptualized and mathematically modeled in future studies.

The birth and death process is described as a common mechanism to explain the dynamic of gene families [54]. Here we propose considering the birth and death process as a fixation and maintenance index, fixation being the probability that a duplicate will spread in a population, and maintenance being the probability that a duplicate is preserved in the long term. This distinction enables us to take into account essential variables such as population size along with functionality of genes.

b) We are tempted to consider duplication as a primary driving force in the adaptive evolution of genomes, but this "master" mutation still has to be integrated into an evolutionary context to assess its importance in genome evolution. Single correlations between duplicate numbers between families or their respective similarities are too scarce to unravel genome evolution, and history of the duplicates has to be clearly integrated into phylogenetic comparative methods [68].

In future studies, phylogenetic comparative methods should be considered as paradigm.

## Competing interests

The authors declare that they have no competing interests.

## Authors' contributions

Both authors planned and wrote the paper.

## Reviewer's comments

### Reviewer's report 1

#### Dr. Etienne Joly, IPBS, UMR CNRS 5098, Toulouse, France

This reviewer provided no comments for publication. The authors are grateful to the reviewer for helpful suggestions.

### Reviewer's report 2

#### Dr. Lutz Walter, German Primate Center, Goettingen, Germany

This informative review discusses mechanisms and consequences of genome evolution through duplications. The authors considered both whole genome and segmental duplications. The evolutionary consequences are discussed and the authors consider favouring as well as opposing mechanisms to fix and maintain such duplications. I have only a minor point concerning the fixation and maintenance of duplicates. This is a central point of the manuscript and, therefore, should deserve more description than just a few sentences. Examples maybe helpful for the readers.

### Author's response

We completely agree with the comment of Dr. Walter about the conceptual distinction between fixation and maintenance of duplicates. In our opinion, this is an essential and critical point to be considered in all modern and future studies based on the fates of duplicates and their role on genomes evolution. In line with the reviewer's suggestion, we now describe more deeply the theoretical and conceptual distinction between each of the steps leading to the generation of preserved gene duplicates. In addition, we discuss one example in which such a distinction is crucial to avoid several potential sources of error and bias in the estimates of evolutionary rates of duplication.

### Reviewer's report 3

#### Dr. W. Ford Doolittle, Dalhousie University, Halifax, Nova Scotia, Canada

This reviewer provided no comments for publication.

## References

[B1] Ohno S Evolution by gene duplication 1970 Springer Verlag

[B2] Lynch M Conery JS The origins of genome complexity Science 2003 302 1401 1404 10.1126/science.1089370 14631042

[B3] Bailey JA Gu Z Clark RA Reinert K Samonte RV Schwartz S Adams MD Myers EW Li PW Eichler EE Recent segmental duplications in the human genome Science 2002 297 1003 1007 10.1126/science.1072047 12169732

[B4] Bailey JA Eichler EE Primate segmental duplications: crucibles of evolution, diversity and disease Nature Reviews Genetics 2006 7 552 564 10.1038/nrg1895 16770338

[B5] Kulski JK Gaudieri S Bellgard M Balmer L Giles K Inoko H Dawkins RL The evolution of MHC diversity by segmental duplication and transposition of retroelements Journal of Molecular Evolution 1997 45 599 609 10.1007/PL00006264 9419237

[B6] Salsberg SL White O Peterson J Eisen JA Microbial genes in the human genome: Lateral transfert or gene loss? Science 2001 292 1903 1906 10.1126/science.1061036 11358996

[B7] Hansen MT Multiplicity of genome equivalents in the radiation-resistant bacterium *Micrococcus radiodurans* Journal of Bacteriology 1978 134 71 75 649572 10.1128/jb.134.1.71-75.1978 PMC222219

[B8] Otto SP Whitton J Polyploid incidence and evolution Annual Reviews of Genetics 2000 34 401 437 10.1146/annurev.genet.34.1.401 11092833

[B9] Tobiason DM Seifert HS The obligate human pathogen, *Neisseria gonorrhoeae*, is polyploid Plos Biology 2006 4 1069 1078 10.1371/journal.pbio.0040185 PMC1470461 16719561

[B10] Huftona AL Panopouloua G Polyploidy and genome restructuring: a variety of outcomes Current Opinion in Genetics & Development 2009 19 600 606 10.1016/j.gde.2009.10.005 19900800

[B11] Henry IM Dilkes BP Young K Watson B Wu H Comai L Aneuploidy and genetic variation in the Arabidopsis thaliana triploid response Genetics 2005 170 1979 1988 10.1534/genetics.104.037788 15944363 PMC1449780

[B12] Ramsey J Schemske DW Pathways, mechanisms and rates of polyploid formation in flowering plants Annual Review of Ecology and Systematics 1998 29 467 501 10.1146/annurev.ecolsys.29.1.467

[B13] Ramsey J Schemske DW Neopolyploidy in flowering plants Annual Review of Ecology and Systematics 2002 33 589 639 10.1146/annurev.ecolsys.33.010802.150437

[B14] Kondrashov FA Kondrashov AS Role of selection in fixation of gene duplications J Theor Biol 2006 239 141 151 10.1016/j.jtbi.2005.08.033 16242725

[B15] Davis JC Petrov DA Preferential duplication of conserved proteins in eukaryotic genomes PLOS Biology 2004 2 318 326 10.1371/journal.pbio.0020055 PMC368158 15024414

[B16] Pan D Zhang L Quantifying the major mechanisms of recent gene duplications in the human and mouse genomes: a novel strategy to estimate gene duplication rates Genome Biol 2007 8 R158 10.1186/gb-2007-8-8-r158 17683522 PMC2374989

[B17] Jensen RA Enzyme recruitment in evolution of new function Annual Review of Microbial 1976 30 409 425 10.1146/annurev.mi.30.100176.002205 791073

[B18] Hughes AL The evolution of functionally novel proteins after gene duplication Proc R Soc Lond B Biol Sci 1994 256 119 124 10.1098/rspb.1994.0058 8029240

[B19] Force A Lynch M Pickett FB Amores A Yan YL Postlethwait J Preservation of duplicate genes by complementary, degenerative mutations Genetics 1999 151 1531 1545 10101175 10.1093/genetics/151.4.1531 PMC1460548

[B20] Lynch M Force A The Probability of Duplicate Gene Preservation by Subfunctionalization Genetics 2000 154 459 473 10629003 10.1093/genetics/154.1.459 PMC1460895

[B21] Lynch M O'Hely M Walsh B Force A The probability of preservation of a newly arisen gene duplicate Genetics 2001 159 1789 1804 11779815 10.1093/genetics/159.4.1789 PMC1461922

[B22] He X Zhang J Rapid subfunctionalization accompanied by prolonged and substantial neofunctionalization in duplicate gene evolution Genetics 2005 169 1157 1164 10.1534/genetics.104.037051 15654095 PMC1449125

[B23] Levasseur A Orlando L Bailly X Milinkovitch MC Danchin EG Pontarotti P Conceptual bases for quantifying the role of the environment on gene evolution: the participation of positive selection and neutral evolution Biological Reviews Camb Philos Soc 2007 82 551 572 10.1111/j.1469-185X.2007.00024.x 17944617

[B24] van Hoof A Conserved functions of yeast genes support the duplication, degeneration and complementation model for gene duplication Genetics 2005 171 1455 1461 10.1534/genetics.105.044057 15965245 PMC1456075

[B25] Song K Lu P Tang K Osborn TC Rapid genome change in synthetic polyploids of *Brassica *and its implications for polyploid evolution Proceedings of the National Academy of Sciences of the United States of America 1995 92 7719 7723 10.1073/pnas.92.17.7719 7644483 PMC41217

[B26] Adams KL Evolution of duplicate gene expression in polyploid and hybrid plants Journal of heredity 2007 98 136 141 10.1093/jhered/esl061 17208934

[B27] Chen ZJ Ni Z Mechanisms of genomic rearrangements and gene expression changes in plant polyploids Bioessays 2006 28 240 252 10.1002/bies.20374 16479580 PMC1986666

[B28] Josefsson C Dilkes B Comai L Parent-dependent loss of gene silencing during interspecies hybridization Curr Biol 2006 16 1322 8 10.1016/j.cub.2006.05.045 16824920

[B29] Bennett RJ Johnson AD Completion of a parasexual cycle in Candida albicans by induced chromosome loss in tetraploid strains EMBO J 2003 22 2505 15 10.1093/emboj/cdg235 12743044 PMC155993

[B30] Gerstein AC Chun HJ Grant A Otto SP Genomic convergence toward diploidy in *Saccharomyces cerevisiae* PLoS Genet 2006 2 e145 10.1371/journal.pgen.0020145 17002497 PMC1570378

[B31] Salmon A Ainouche M Wendel JF Genetic and epigenetic consequences of recent hybridization and polyploidy in *Spartina *(*Poaceae*) Molecular Ecology 2005 14 1163 1175 10.1111/j.1365-294X.2005.02488.x 15773943

[B32] Udall JA Swanson JM Nettleton D Percifield RJ Wendel JF A novel approach for characterizing expression levels of genes duplicated by polyploidy Genetics 2006 173 1823 7 10.1534/genetics.106.058271 16702424 PMC1526680

[B33] Albertin W Balliau T Brabant P Chèvre AM Eber F Malosse C Thiellement H Numerous and rapid nonstochastic modifications of gene products in newly synthesized *Brassica napus *allotetraploids Genetics 2006 173 1101 1113 10.1534/genetics.106.057554 16624896 PMC1526534

[B34] Albertin W Brabant P Catrice O Eber F Jenczewski E Chèvre AM Thiellement H Autopolyploidy in cabbage (*Brassica oleracea L*.) does not alter significantly the proteomes of green tissues Proteomics 2005 5 2131 2139 10.1002/pmic.200401092 15852348

[B35] Dykhuizen DE Hartl DL Functional effects of PGI allozymes in *Escherichia coli* Genetics 1983 105 1 18 6352406 10.1093/genetics/105.1.1 PMC1202137

[B36] Kimura M The neutral theory of molecular evolution 1983 Cambridge University Press, Cambridge UK

[B37] True JR Carroll SB Gene co-option in physiological and morphological evolution Annu Rev Cell Dev Biol 2002 18 53 80 10.1146/annurev.cellbio.18.020402.140619 12142278

[B38] Danchin EG Pontarotti P Towards the reconstruction of the bilaterian ancestral pre-MHC region Trends Genet 2004 20 587 91 10.1016/j.tig.2004.09.009 15522451

[B39] Hancock JM Gene factories, microfunctionalization and the evolution of gene families Trends in Genetics 2005 21 591 595 10.1016/j.tig.2005.08.008 16153739

[B40] Hendrickson H Slechta ES Bergthorsson U Andersson DI Roth JR Amplification-mutagenesis: evidence that "directed" adaptive mutation and general hypermutability result from growth with a selected gene amplification Proc Natl Acad Sci USA 2002 99 2164 9 10.1073/pnas.032680899 11830643 PMC122336

[B41] Francino MP An adaptive radiation model for the origin of new gene functions Nature Genetics 2005 37 573 577 10.1038/ng1579 15920518

[B42] Rastogi S Liberles DA Subfunctionalization of duplicated genes as a transition state to neofunctionalization BMC Evolutionary Biology 2005 5 28 10.1186/1471-2148-5-28 15831095 PMC1112588

[B43] Wagner A Robustness and evolvability: A paradox resolved Proc Roy Soc London Series B 2008 275 91 100 10.1098/rspb.2007.1137 PMC2562401 17971325

[B44] Lynch M Conery JS The evolutionary fate and consequences of duplicate genes Science 2000 290 1151 1155 10.1126/science.290.5494.1151 11073452

[B45] Cooke J Nowak MA Boerlijst M Maynard-Smith J Evolutionary origins and maintenance of redundant gene expression during metazoan development Trends in Genetics 1997 13 360 364 10.1016/S0168-9525(97)01233-X 9287491

[B46] Veitia RA Exploring the etiology of haploinsufficiency Bioessays 2002 24 175 184 10.1002/bies.10023 11835282

[B47] Veitia Reiner A Gene Dosage Balance in Cellular Pathways Implications for Dominance and Gene Duplicability Genetics 2004 168 569 574 10.1534/genetics.104.029785 15454568 PMC1448121

[B48] Papp B Pal C Hurst LD Dosage sensitivity and the evolution of gene families in yeast Nature 2003 424 194 197 10.1038/nature01771 12853957

[B49] Wapinski I Pfeffer A Friedman N Regev A Natural history and evolutionary principles of gene duplication in fungi Nature 2007 449 54 61 10.1038/nature06107 17805289

[B50] Darbo E Danchin EG Mc Dermott MF Pontarotti P Evolution of major histocompatibility complex by "en bloc" duplication before mammalian radiation Immunogenetics 2008 60 423 38 10.1007/s00251-008-0301-7 18560826

[B51] Britten RJ Almost all human genes resulted from ancient duplication Proc Natl Acad Sci USA 2006 103 19027 32 10.1073/pnas.0608796103 17146051 PMC1748171

[B52] Dehal P Boore JL Two rounds of whole genome duplication in the ancestral vertebrate PLoS Biol 2005 3 e314 10.1371/journal.pbio.0030314 16128622 PMC1197285

[B53] Cotton JA Page RDM Rates and patterns of gene duplication and loss in the human genome Proceedings Biological sciences/The Royal Society 2005 272 277 283 10.1098/rspb.2004.2969 15705552 PMC1634978

[B54] Nei M Rooney AP Concerted and birth-and-death evolution of multigene families Annu Rev Genet 2005 39 121 52 10.1146/annurev.genet.39.073003.112240 16285855 PMC1464479

[B55] Hahn MW De Bie T Stajich JE Nguyen C Cristianini N Estimating the tempo and mode of gene family evolution from comparative genomic data Genome Res 2005 15 1153 1160 10.1101/gr.3567505 16077014 PMC1182228

[B56] Su AI Cooke MP Ching KA Hakak Y Walker JR Wiltshire T Orth AP Vega RG Sapinoso LM Moqrich A Patapoutian A Hampton GM Schultz PG Hogenesch JB Large-scale analysis of the human and mouse transcriptomes Proc Natl Acad Sci 2002 99 4465 4470 10.1073/pnas.012025199 11904358 PMC123671

[B57] Garland T Jr Bennett AF Rezende EL Phylogenetic approaches in comparative physiology Journal of Experimental Biology 2005 208 3015 3035 10.1242/jeb.01745 16081601

[B58] Doxey AC Yaish MW Moffat BA Griffith M McConkey BJ Functional divergence in the *Arabidopsis *beta-1,3-glucanase gene family inferred by phylogenetic reconstruction of expression states Molecular biology and evolution 2007 24 1045 1055 10.1093/molbev/msm024 17272678

[B59] Hittinger CT Carroll SB Gene duplication and the adaptive evolution of a classic genetic switch Nature 2007 449 677 681 10.1038/nature06151 17928853

[B60] Hickman MA Rusche LN Substitution as a mechanism for genetic robustness: the duplicated deacetylases Hst1p and Sir2p in *Saccharomyces cerevisiae* PLoS Genetics 2007 3 e126 10.1371/journal.pgen.0030126 17676954 PMC1937012

[B61] Cusack BP Wolfe KH When gene marriages don't work out: divorce by subfunctionalization Trends in Genetics 2007 23 270 272 10.1016/j.tig.2007.03.010 17418444

[B62] Zhang JZ Parallel adaptive origins of digestive RNases in Asian and African leaf monkeys Nature Genetics 2006 38 819 823 10.1038/ng1812 16767103

[B63] Scannell DR Butler G Wolfe KH Yeast genome evolution--the origin of the species Yeast 2007 24 929 42 10.1002/yea.1515 17621376

[B64] Brunet FG Roest Crollius H Paris M Aury JM Gibert P Jaillon O Laudet V Robinson-Rechavi M Gene loss and evolutionary rates following whole-genome duplication in teleost fishes Mol Biol Evol 2006 23 1808 16 10.1093/molbev/msl049 16809621

[B65] Kellis M Birren BW Lander ES Proof and evolutionary analysis of ancient genome duplication in the yeast *Saccharomyces cerevisiae* Nature 2004 428 617 24 10.1038/nature02424 15004568

[B66] Tirosh I Barkai N Comparative analysis indicates regulatory neofunctionalization of yeast duplicates Genome Biology 2007 8 R50 10.1186/gb-2007-8-4-r50 17411427 PMC1895995

[B67] Marques AC Vinckenbosch N Brawand D Kaessmann H Functional diversification of duplicate genes through subcellular adaptation of encoded proteins Genome Biol 2008 9 R54 10.1186/gb-2008-9-3-r54 18336717 PMC2397506

[B68] Harvey PH Pagel MD The Comparative Method in Evolutionary Biology Oxford Series in Ecology and Evolution 1991 239 Oxford University Press, Oxford

